# Heat and Humidity in the City: Neighborhood Heat Index Variability in a Mid-Sized City in the Southeastern United States

**DOI:** 10.3390/ijerph13010117

**Published:** 2016-01-11

**Authors:** Alisa L. Hass, Kelsey N. Ellis, Lisa Reyes Mason, Jon M. Hathaway, David A. Howe

**Affiliations:** 1 Department of Geography, The University of Tennessee, 304 Burchfiel Geography Building, Knoxville, TN 37996, USA; ellis@utk.edu; 2 College of Social Work, The University of Tennessee, 408 Henson Hall, Knoxville, TN 37996, USA; mason@utk.edu; 3 Department of Civil and Environmental Engineering, The University of Tennessee, 325 John D. Tickle Building, Knoxville, TN 37996, USA; hathaway@utk.edu (J.M.H.); dhowe@vols.utk.edu (D.A.H.)

**Keywords:** urban heat island, heat exposure, microclimate, impervious surface, canopy

## Abstract

Daily weather conditions for an entire city are usually represented by a single weather station, often located at a nearby airport. This resolution of atmospheric data fails to recognize the microscale climatic variability associated with land use decisions across and within urban neighborhoods. This study uses heat index, a measure of the combined effects of temperature and humidity, to assess the variability of heat exposure from ten weather stations across four urban neighborhoods and two control locations (downtown and in a nearby nature center) in Knoxville, Tennessee, USA. Results suggest that trees may negate a portion of excess urban heat, but are also associated with greater humidity. As a result, the heat index of locations with more trees is significantly higher than downtown and areas with fewer trees. Trees may also reduce heat stress by shading individuals from incoming radiation, though this is not considered in this study. Greater amounts of impervious surfaces correspond with reduced evapotranspiration and greater runoff, in terms of overall mass balance, leading to a higher temperature, but lower relative humidity. Heat index and relative humidity were found to significantly vary between locations with different tree cover and neighborhood characteristics for the full study time period as well as for the top 10% of heat index days. This work demonstrates the need for high-resolution climate data and the use of additional measures beyond temperature to understand urban neighborhood exposure to extreme heat, and expresses the importance of considering vulnerability differences among residents when analyzing neighborhood-scale impacts.

## 1. Introduction

Heat waves, such as those recently experienced in Karachi, Pakistan, in 2015 where more than 1000 people succumbed to heat-related deaths [1], and Europe in 2003 which resulted in thousands of deaths [2], are becoming more frequent with changing climate [3,4,5,6]. For those who live in highly populated areas, there is a higher risk of experiencing a more extreme heat wave than for those who live in surrounding areas because of the Urban Heat Island (UHI) effect [7,8]. The purpose of this project is to investigate how environmental conditions relevant to human heat stress vary across and within four urban neighborhoods in a mid-sized city using heat index, a measure of the combined effect of temperature and humidity. Heat index variability is related to neighborhood and local-scale differences in percent impervious surface cover and percent vegetated cover.

### 1.1. The Urban Heat Island

The UHI effect is a well-studied phenomena where densely populated areas observe higher temperatures than more sparsely populated areas, especially at night [7,8,9,10,11,12]. This phenomena exists primarily because of decreased albedo from a greater concentration of absorptive surfaces than found in rural areas [13]; decreased evapotranspiration because of a lack of vegetation and high levels of rainfall partitioning to runoff; increased heat capacity from asphalt, concrete, steel, glass, and other manmade materials [13]; the geometry of the built environment causing radiation trapping, wind disturbance, and increased albedo when compared to flat surfaces [13]; and an increase in heating from anthropogenic activities, such as using air conditioners, generators, and cars [10,14,15,16,17]. Additional differences have been observed between not only urban and rural comparisons [16] but also between urban neighborhoods, where neighborhood characteristics (e.g., density of housing, amount of vegetation) affect UHI intensity [4,11,18]. Therefore, UHI intensity will vary between and within cities, and results from a study in one city may not sufficiently inform another.

The UHI is related to the socioeconomic characteristics of an area, as locations with lower income are more likely to have higher population densities, less green space, and less access to resources like air conditioning [11,19,20,21,22,23,24]. Further, the UHI is expected to intensify with changing climate, creating an increase in days with high heat stress in both rural and urban areas, indicating that populations in both rural and urban settings will be more vulnerable to heat stress [17], potentially compounding the effects of UHI on urban populations.

### 1.2. Assessing UHI and Thermal Comfort

To assess the intensity and locality of UHI effects and, in turn, determine the best way to mitigate excessive heat and reduce the risk of heat exposure within an urban location, small-scale, high-resolution data must be collected and analyzed. Meso-scale studies of UHI, covering cities, states, and even whole countries [25], have been undertaken using simulations [26] and modeling [17], remote sensing [2,22,27,28], census data [22,27], and preexisting city-wide weather station data [5]. These studies often produce an understanding of UHI over a large spatial area that can provide comparison data on urban sprawl in cities, and between cities and regions, and help to understand climatic changes through time [2,5].

Alternatively, local-scale UHI studies involving neighborhood-level data [25] given by numerous weather stations [29] or remotely sensed data [12,30,31] provide information about the difference in weather conditions, such as maximum temperature, experienced by those within a single city based on the proportion of green space, building density and configuration, and quantity of impervious surfaces within their neighborhood. Results from such studies suggest that inner cities typically experience a higher temperature and suburbs exhibit a lower temperature [11,32,33]. This fine-scale research reveals correlations between increased UHI strength and neighborhoods that are more densely populated, have lower socioeconomic status, and high have concentrations of racial and ethnic minorities [11,21,22]. Given this information, microscale research can be conducted to determine how small scale factors (e.g., living conditions, building types, access to resources) will impact a specific individual.

These microscale studies of UHI have drawn attention to the variability of individually experienced temperatures by using mortality records [33] or small wearable sensors designed to measure air temperature around each person [34]. This type of microscale study has also been carried out through surveys in which selected individuals provided information on their living situation as well as how much time was spent in their living quarters and on their daily habits, such as washing dishes, to provide data for a formula that could be used to assign a dampness and mold factor to each home and assess differences between gender and age groups [35]. As individuals typically alternate between indoors and outdoors throughout the day, the amount of time spent in either varies between persons, resulting in a variable level of heat exposure [36].

The representative proxy for determining the existence and intensity of UHI is often temperature collected from one or more *in*-*situ* weather stations, such as those near airports [9,12,22,34,37,38,39], with the classic UHI signal demonstrating highest temperatures in the areas with the highest populations, building density, and anthropogenic activities. However, the impact of high temperatures on humans is increased with increased moisture in the air [40]. High moisture content decreases a person’s ability to evaporate sweat off the skin, and thus decreases the effectiveness of the body’s natural cooling system [41]. Often inner cities have a lower humidity than surrounding areas as there is less vegetation and a large amount of rainfall partitioned to runoff due to impervious surfaces, resulting in a slightly lower heat index [7] and potentially lower heat stress on humans [11]. Vegetation cools surrounding air through transpiration and corresponding evaporative cooling [9]. The shading provided by trees will also affect the human energy budget, which takes into account metabolism, net radiation, latent heat flux from respiration and sweat, and convective heat transfer, among other factors, during times of high temperatures by blocking some direct radiation [42,43]. Heat indices are calculated for the combined effect of temperature and humidity and used to determine the “feels like” outdoor temperature [44] and to issue heat warnings to the public when necessary [45].

In an effort to include humidity into the understanding of the climate of intra-urban neighborhoods and for individuals residing in an urban area, thermal comfort has been assessed using mean monthly relative humidity and air temperature [46]. Multiple studies have used the Humidex variable, which includes both human physiological variables (*i.e.*, age, gender, clothing, activities) and environmental variables (*i.e.*, wind, insolation, temperature, relative humidity), and other thermal indices to determine how the influence of humidity on high temperatures is experienced at a finer scale [46,47]. These studies have concluded that relative humidity will impact (albeit variably, based on temperature) human thermal comfort, especially during times of warm conditions, such as mid-day, in intra-urban settings [46,48]. Additionally, UHI intensity and variability throughout an urban area were greater when using thermal indices instead of temperature [48]. Studies on individually experienced temperature (e.g., Harlan *et al.* [11]) report that humidity is one of many variables that influence thermal comfort.

### 1.3. Urban Health

The UHI may affect the health of those living in an urban area. Heat-related illnesses and deaths tend to increase in urban areas during heat waves, especially in areas that are unaccustomed to high temperatures like the upper mid-west, USA [4,6,20,49,50,51]. Despite acclimation to potential extreme weather such as heat waves, the overall increased exposure to extreme heat in the southern United States, coupled with an aging population, results in an overall higher rate of heat-related deaths [52]. According to Harlan *et al.* [11] (p. 2848), heat inequality is a serious issue with the “highest morbidity and mortality associated with extreme heat appear[ing] to occur in cities and fall[ing] disproportionately upon marginalized groups: the poor, minorities, and elderly”. Access to resources, such as central air conditioning, often varies with race and other socioeconomic characteristics, placing certain groups at greater disadvantage [53,54,55]. The type of dwelling that a person lives in can also result in higher indoor temperatures; for instance, a top floor of an apartment is often warmer than a house [56].

Indirectly, high temperatures may make health issues, such as asthma, air pollution, and allergens, worse especially in populations more readily exposed to high heat [6,17,57]. Some inter-city population-level studies have shown that humidity does not affect health as much as temperature; however other factors (e.g., sample size) might mask the significance of humidity [58]. Additionally, humidity can result in more severe consequences at the intra-city neighborhood level, especially where there is an increased population of those with a lessened ability to thermoregulate (e.g., elderly populations) [59]. The combined effect of UHI, a lack of resources, and less coping ability may place these populations at a greater risk of suffering heat-related stress, illness, and mortality. It is important to note that studies that measure individually experienced temperatures, like those mentioned above, are needed to understand individual health risk and how this risk is related to UHI [11,34,59]. With the expected changing of climate towards more heat waves, these vulnerable populations and those that are not acclimated to high heat will become more at risk to heat illness and stress [51].

### 1.4. Assessing the Role of Urban Neighborhood Characteristics in Knoxville, Tennessee on Heat Index

This work utilizes neighborhood-scale data to determine how the daily 1500 LDT heat index (HI) varies within and between four diverse urban neighborhoods in Knoxville, TN, USA, as well as at control locations in downtown Knoxville and at Ijams Nature Center. Within each neighborhood, we compare the HI of two different locations with varying levels of tree cover and impervious surfaces, and then make comparisons between neighborhoods. We additionally analyze the top 10% of HI values to determine if there is greater HI variability at 1500 LDT during the warmest days of the study period. The significance of this work is using strategically located weather stations to estimate the variability of combined exposure of the effect of humidity on temperatures during the warm season across surface and socioeconomic characteristics.

## 2. Materials and Methods

### 2.1. Site Descriptions and Data Collection

Data were collected from 10 identical weather stations for a period of one year in Knoxville, Tennessee, USA. The City of Knoxville, the third largest in the state, had an estimated population of 184,281 people on 1 July 2014 [60]. The Knoxville Metropolitan Statistical Area, which encompasses the entirety of the study location, contained an estimated 837,571 people on 1 July 2014, with an increase of about 20,000 people within the prior four years [61]. In 2010, it was estimated that Knoxville covers 98.52 mi^2^ with an average population density of 1815.6 persons per mi^2^ [60].

Knoxville is located in eastern Tennessee, in a valley between the Cumberland Plateau to the west and the foothills of The Great Smoky Mountains National Park to the east. Knoxville experiences a climate categorized as humid subtropical, with warm summers exemplified by an average maximum temperature of 31.2 °C in July and cool winters exemplified with an average maximum temperature of 8.5 °C in January [62]. As the National Weather Service classifies heat indices above 26.7 °C and 40% humidity as hazardous [63] we chose the warmest five months of the year (May, June, July, August, September) for the purposes of this study as they hold the highest potential for cautionary levels (or above) during a normal year.

**Figure 1:**
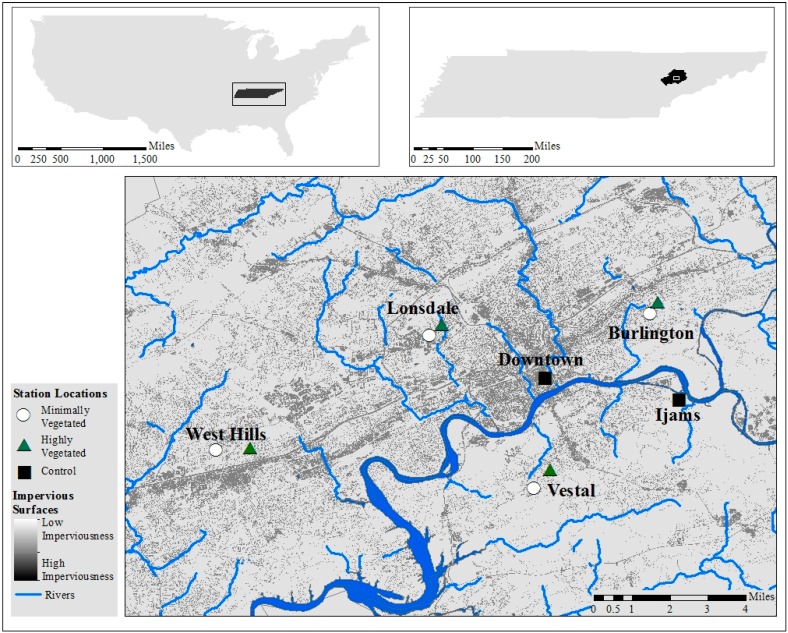
Locations of minimally vegetated (MV) and highly vegetated (HV) weather stations within each neighborhood, and control weather stations downtown and in Ijams Nature Center, and amount of imperviousness in the City of Knoxville.

Weather stations were positioned in four urban neighborhoods, downtown Knoxville, and Ijams Nature Center (Figure 1). Each of the four neighborhoods (Burlington, Lonsdale, Vestal, and West Hills) contains one station in a minimally vegetated (MV) location (very little vegetation) and one station in a highly vegetated (HV) location (more dense vegetative cover). These specific urban neighborhoods were chosen as they are all within the Knoxville city limits, they provided a geographic coverage of the city from all cardinal directions, and they allowed for the exploration of different socioeconomic characteristics that vary throughout the city as described below. Site selection was undertaken carefully to ensure similar elevations in each neighborhood location as well as to avoid localized weather influences, such as cold air drainage. Locating weather stations in both MV and HV areas of each urban neighborhood allows examination of how heat exposure may vary in a small area and can help provide an understanding of how vegetation can impact relative humidity and therefore HI in each neighborhood.

The four neighborhoods were chosen to reflect a range of socioeconomic characteristics (Table 1). Lonsdale has the highest population density and lowest mean income, and West Hills has the lowest population density and the highest mean income [64]. By race and ethnicity, the populations in West Hills and Vestal are predominantly White. In Burlington, most residents are African American. In Lonsdale, residents are more racially and ethnically diverse, including White, African American, and Hispanic households [64].

**Table 1:** Population density, approximate mean income, and general qualitative description of four Knoxville, Tennessee neighborhoods examined in this study [64].

Neighborhood	Population Density (People/sq km)	Approximate Mean Income (USD)	Qualitative Description
Lonsdale	5941	22,950	Medium density housing with parks and open space
Burlington	4971	29,447	Medium density housing with parks and open space
Vestal	3322	24,456	Medium density housing with parks, open space, and shopping centers
West Hills	2052	42,147	Medium density housing with parks, open space, and a large amount of shopping centers and highway access

The weather stations located in the control locations (one each in downtown Knoxville and Ijams Nature Center) serve as a comparison to typical UHI patterns seen in urban areas, that is, higher temperatures and lower humidity being experienced in downtown locations than in vegetated and sparsely developed areas. These locations also serve to compare the traditional UHI temperature patterns across the city to the patterns of HI.

The temperature and relative humidity were measured in five-minute increments at each weather station. The stations consist of Onset Smart Sensors attached to a Cantex Junction Box (20 × 20 × 10 cm). The sensors are connected to a HOBO Micro Station Data Logger (H21-002). Temperature measurements by the Onset 12-bit T/RH Smart Sensor (S-THB-M002) have an accuracy of ±0.21 °C, and a resolution of 0.02 °C. Relative humidity measurements have a range of 0%–100%, with an accuracy of ±2.5% and a resolution of 0.1%. The manufacturer establishes accuracy for the weather stations based on testing of the components prior to shipment. Additionally, before deployment, the weather stations were tested to ensure consistent readings across all units. The sensors are installed approximately 2.25 m above ground inside of a white, vented enclosure. Ideally, data would be collected at a lower height, but this height was chosen to minimize vandalism.

### 2.2. Data

The stations began collecting data on 2 July 2014. This study analyzed data from 2 July 2014 through 30 September 2014 and 1 May 2015 through 1 July 2015. This data set covered a full warm season in Knoxville. Because of an act of vandalism at the Lonsdale MV location, data were not available for this location from 15–21 August 2014. Additionally, data sensor malfunctions resulted in data unavailability from 1–5 May at the Lonsdale MV station, 1–4 May 2015 at the West Hills HV station, and 1–5 May 2015 at the Burlington HV station.

**Figure 2:**
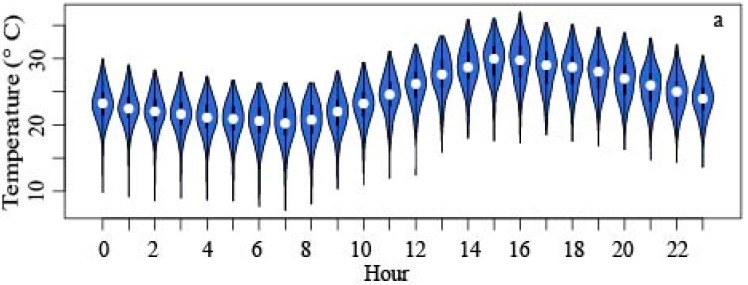

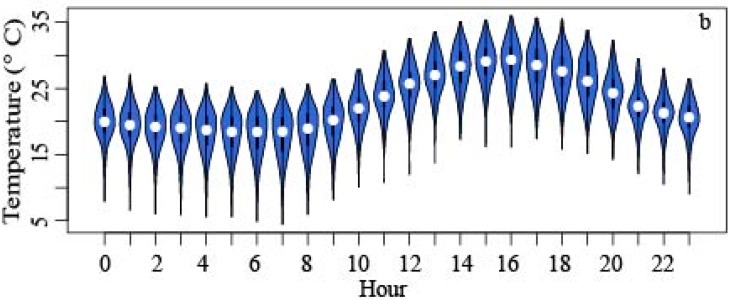
Hourly temperature distributions for Downtown (**a**) and Ijams (**b**) during the study period, including hour of the day and temperature.

Daily temperature (T) and relative humidity (RH) were parsed from each station’s data at 1500 LDT daily. The 1500 LDT observation was chosen since the hottest time of day typically occurs around this time because of a delay in insolation reaching and warming the surface [65]. Additionally, hourly temperature distributions at the control locations for the study period indicate the highest daily temperature is typically between 1500 LDT and 1600 LDT (Figure 2). Choosing the hottest time of the day targets the HI during maximum heating for each day during the warm season. This was confirmed with preliminary unadjusted hourly heat index distributions at our control locations for the time period studied, with the maximum heat index occurring at both locations between 1400 LDT and 1500 LDT though heat index showed a larger overall variation than temperature. Keeping a consistent observation time allows for snapshot comparisons across the city at the approximate time of maximum heat loading. Changes in this daily pattern due to synoptic forcing were not considered in this study.

### 2.3. Data Processing

#### 2.3.1. Calculation of Imperviousness and Tree Cover

The amount of impervious surface and tree cover within 100 m of each weather station were quantified. The 100 m radius was chosen because land cover has greatest influence on air temperature at radii less than 500 m, with the effects diminishing at larger distances [66,67]. Stewart and Oke [68] suggest a 100–200 m circle of influence for their “local climate zones”. Similarly, Gallo *et al.* [69] and Li and Roth [70] found that 100 m radii were the ideal spatial resolution for visualizing land use effects on diurnal temperature range and UHI intensity, respectively.

GIS data for impervious cover in Knox County were obtained from KGIS (kgis.org), a Geographic Information System collaboration between the city of Knoxville, Knox County, and Knoxville Utilities. The 1-m solution raster was processed with ArcMap 10.2. Every impervious cell is represented by a value of 1 and every other cell is represented by a value of 0. The percentage of impervious cover for a given area was calculated by dividing the total number of impervious cells by the total area.

Tree cover within the 100-m radius of each weather station was estimated using i-Tree Canopy, an online analysis tool by the USDA Forest Service. I-Tree Canopy uses aerial images available in Google Maps to produce an estimate of tree cover. Project boundaries (the 100-m radius of each weather station) were loaded from ArcMap 10.2 for each area. Random sample points were generated by i-Tree Canopy and classified by the user as either “tree” or “non-tree”. For the classification of an entire city, i-Tree suggests using between 500 and 1000 survey points; 300 survey points were classified within each of the 100-m radii. These analyses were completed three times and the average result was used.

#### 2.3.2. Calculation of Heat Index

HI was calculated from daily 1500 LDT T and RH data using the NOAA Rothfusz equation [63,71], which is based on T readings in °F, as follows:

(1) $$\begin{array}{ll} HI=\;-42.379+ & 2.04901523T+10.14333127R-0.22475541TR-6.83783\\ & *10^{-3}T^{2}-5.481717*10^{-2}R^{2}+1.22874*10^{-3}T^{2}R+8.5282\\ & *10^{-4}TR^{2}-1.99*10^{-6}T^{2}R^{2} \end{array}$$

For days when T was between 80–87 °F and RH was greater than 85%, the below adjustment was added to the Rothfusz regression equation [63]:

(2) $$Adjustment=\left[\frac{RH-85}{10}\right]*\left[\frac{87-T}{5}\right]$$

The Rothfusz regression equation is not suitable to use when the HI is below 80 °F [63]. For all days in which the Rothfusz regression equation yielded a HI of less than 80 °F, the following equation was used to recalculate the HI [63]:

(3) HI=0.5*{T+61.0+[(T−68.0)*1.2]+(RH*0.094)}

The results of the above equation were averaged with T to obtain the final HI for days with HI below 80 °F [63]. All temperature and HI data were converted from Fahrenheit to Celsius prior to statistical analysis.

#### 2.3.3. Statistical Analysis

T, RH, and HI were used to estimate heat and humidity variability across study neighborhoods during the warm season. First, HI, T, and RH were compared between HV and MV locations within each of the four urban neighborhoods. Second, HI was compared across neighborhoods (averaged HV and MV station data) and the control locations (downtown Knoxville and Ijams). Paired-sample *t*-tests were used for both of these analyses, except in the case of missing data where independent *t*-tests were used. We address the multiple comparison problem, where using a large number of independent *t*-tests could increase the number of tests deemed significant by chance, by verifying our *t*-test results through two-way analysis of variance (ANOVA) tests. Two-way ANOVA tests were used to determine if significance existed between locations during the top 10% of HI values for each station. A two-way ANOVA tests the separate influence of each independent variable, and also analyzes the interaction between the two variables. ANOVA was also specifically used to determine the combined effects of neighborhood and tree cover. Three two-way ANOVAs were performed for each dependent variable (T, RH, HI) to assess the independent and combined influence of neighborhood and tree cover.

## 3. Results and Discussion

### 3.1. Descriptive Statistics

Station characteristics (location, elevation, *etc.*) are listed in Table 2, along with average T, RH, and HI during the study period. Sample size is provided and is based on the number of days available for testing after station malfunction and vandalism data were removed. Mean T and mean RH show the typical UHI pattern of warm and dry conditions in the downtown control location and cool and humid conditions in the Ijams Nature Center control location. Mean HI was highest downtown and at Ijams; lowest mean HI was in the HV locations of the four urban neighborhoods. Testing for significant differences between these values is discussed in Section 3.3, Section 3.4, Section 3.5, Section 3.6 and Section 3.7. The locations with the highest variation in HI during the warm season include Ijams and the HV station in West Hills (in order, respectively).

**Table 2:** Station information, including neighborhood name and station designation, elevation (m), location (° latitude and longitude), and sample size (number of days recorded). Also shown for each station is the 1500 LDT mean temperature (°C), mean relative humidity (%), mean heat index (°C), maximum heat index (°C), minimum heat index (°C), and the standard deviation of the heat index (°C).

Neighborhood	Lonsdale		West Hills		Vestal		Burlington		Downtown	Ijams
Station Designation	MV	HV	MV	HV	MV	HV	MV	HV		
Elevation (m)	290.8	293.7	316.4	312.2	288.5	280.4	355.9	316.1	286.4	290.0
Latitude	35.980	35.984	35.936	35.937	35.922	35.929	35.988	35.993	35.964	35.956
Longitude	−83.962	−83.957	−84.043	−84.030	−83.922	−83.916	−83.878	−83.875	−83.918	−83.866
Sample Size	145	153	153	148	153	153	153	148	153	153
T	28.73	28.38	28.16	27.74	28.94	28.56	28.69	27.56	29.66	28.77
RH	53.61	55.62	58.14	63.00	55.68	57.31	56.46	59.62	51.52	62.46
HI	29.94	29.79	29.81	29.61	30.63	30.42	30.32	28.86	31.12	31.46
Max HI	37.98	39.24	39.3	39.82	41.17	40.52	40.94	36.94	40.98	43.62
Min HI	16.32	16.60	18.48	18.01	16.78	19.60	16.62	17.66	17.17	18.14
HI Standard Deviation	4.12	4.18	4.38	4.70	4.40	4.46	4.47	3.92	4.14	4.86

### 3.2. Imperviousness and Tree Cover

Percent of imperviousness and tree canopy at each weather station (Table 3, also visualized in Figure 1) were estimated to demonstrate the appropriateness of station location. Within each individual neighborhood, the MV location showed a higher level of imperviousness than the corresponding HV location. The greatest amount of neighborhood imperviousness is 48.8% at the Lonsdale MV station. Likewise, the HV station has a higher level of tree cover than the corresponding MV station for each neighborhood, as desired per the experimental plan. The greatest amount of tree cover is 51.3% at Vestal. The downtown station showed the highest level of impervious surfaces (84%) and the lowest amount of tree cover (4.6%). The station located within Ijams Nature Center showed the highest amount of tree cover (78.8%) and lowest amount of impervious land cover (3.6%). Standard error for these calculations never exceeded 3% using 300 survey points.

The amount of impervious surfaces and tree cover can affect both the T and RH of an area, which in turn can affect the HI. Greater imperviousness corresponds with reduced evapotranspiration and greater rainfall lost to runoff (*i.e.*, moisture within the watershed), leading to a higher T and lower RH [13]. Increased tree cover reduces T by shading the area from incoming solar radiation and increasing evapotranspiration, leading to a higher RH [10,13,72,73]. Their combined impact on local HI is subject to analysis in Section 3.6.

**Table 3:** Percent impervious land cover and tree cover within a 100-m radius of each station.

Neighborhood	Station Designation	Imperviousness	Tree Cover
Lonsdale	MV	48.8	7.2
	HV	32.3	28.2
West Hills	MV	23.1	33.0
	HV	20.1	60.1
Vestal	MV	41.7	7.2
	HV	16.9	51.3
Burlington	MV	25.6	27.0
	HV	18.0	47.2
Downtown	−	84.0	4.6
Ijams	−	3.6	78.8

These physical relationships are represented in the data collected here, with the warmest locations occurring at the stations with the greatest amount of impervious surfaces (the MV locations of Lonsdale, West Hills, Vestal, and Burlington) (Table 2). The locations with the highest RH were those with the greatest amount of tree cover (the HV stations of Lonsdale, West Hills, Vestal, and Burlington). The control locations additionally follow the maxim of high T and low RH occurring in areas with high amounts of impervious surfaces and low tree cover (downtown Knoxville) and low T with high RH occurring in areas with low amounts of impervious surfaces and high tree cover (Ijams Nature Center).

### 3.3. Inter-Neighborhood Variability

*T*-tests were used to determine if there are significant differences between the mean HI of the neighborhoods (combined HV and MV station data) and control locations. Vestal and West Hills showed a significantly higher mean HI than the other neighborhoods but were not significantly different from each other (Table 4). Downtown reported a higher HI than all four urban neighborhoods, but this difference is not significant when compared to West Hills (0.48 °C lower) and Vestal (0.59 °C lower). Ijams Nature Center exhibited a significantly higher HI than all other locations, including the neighborhoods and downtown location.

**Table 4:** Comparison of 1500 LDT heat index (°C) between combined data (HV and MV) for each neighborhood and control locations (Downtown and Ijams). Mean differences are shown. Bolded numbers indicate significant results (*p* < 0.05). Negative numbers indicate the column neighborhood is lower than the row neighborhood mean.

Neighborhood	Downtown	Ijams	Lonsdale	West Hills	Vestal	Burlington
Downtown	−	**0.34**	**−1.25**	−0.48	−0.59	**−1.52**
Ijams	**−0.34**	−	**−1.60**	**−0.83**	**−0.82**	**−1.86**
Lonsdale	**1.25**	**1.60**	−	**0.82**	**0.77**	−0.27
West Hills	0.48	**0.83**	**−0.82**	−	−0.11	**−1.03**
Vestal	0.59	**0.82**	**−0.77**	0.11	−	**−0.92**
Burlington	**1.52**	**1.86**	0.27	**1.03**	**0.92**	−

While the T and RH data follow the traditional UHI pattern [13], the HI data highlight the importance of examining combined effects of T and RH. The comparison between the two control locations (downtown and Ijams) gave somewhat unexpected results with the HI being significantly higher at Ijams Nature Center. Typically, downtown locations have higher temperatures because of the UHI effect [13,74], which would seemingly correspond to greater heat exposure. However, because of the consistently higher RH at the Ijams Nature Center, this more than compensates for the lower daytime T, causing a greater HI. The higher RH at Ijams is likely because the greater vegetative cover here leads to increased evapotranspiration, and the vegetation slows air movement and decreases air mixing [13,72,75]. Air mixing is likely different at Ijams than downtown. In the latter, air tunneling and redirection may result in increased mixing of wet and dry air layers, whereas less wind at Ijams might result in less mixing of surface humidity [13]. Although testing this explanation is beyond the study scope, it is a possible area for future research.

Ijams Nature Center also had a significantly higher HI than all of the neighborhoods, likely due to the reasons discussed above. Meanwhile, the downtown location had a higher HI than three of the neighborhoods (statistically significant in Lonsdale and Burlington, *p* < 0.05) likely because of decreased albedo, decreased evapotranspiration, and increased anthropogenic heat sources contributing to the UHI [10,11,12]. Therefore, extremely developed or under-developed land can exhibit higher HIs for different reasons depending on the conditions.

The amount of trees and lack of impervious surfaces in West Hills likely corresponded to the high HI experienced here, but to a lesser degree than Ijams. The cause of the high HI in Vestal is less clear. Vestal has the greatest differences in the impervious surface and tree cover amounts between stations of all neighborhoods, but a significant difference in HI between stations. Perhaps the Vestal locations are each experiencing high HI values for different reasons, the MV station due to increased absorption and daytime heating, and the HV station due to greater humidity. Within-neighborhood differences are discussed in detail in the next section.

Within a city, the UHI strengthens with increased population and building structures and often decreases with income [11]. Given the physical and socioeconomic characteristics of each neighborhood, Lonsdale (West Hills) would be expected to show the highest (lowest) HI because of an increased (decreased) population density and a lower (higher) mean income. West Hills (both MV and HV stations) also has greater tree cover and less imperviousness than most locations, independent of being the HV or MV station. Yet, these results suggest that, in terms of heat index, West Hills’ residents may experience more heat exposure during the warm season. While neighborhood-scale climatic data may imply West Hills residents are more exposed to heat, social data would likely suggest that Lonsdale residents have less resources available for coping with extreme conditions. Indeed, a recent qualitative study in the same four neighborhoods found that Lonsdale, Burlington, and Vestal participants expressed greater concerns about extreme heat, its impacts, and household coping ability than participants from West Hills [76]. Future pairing of physical data, socioeconomic characteristics, and personal experiences of neighborhood residents could expand on these initial insights.

### 3.4. Intra-Neighborhood Variability

*T*-tests were used to determine how HI, T, and RH vary between MV and HV locations within each urban neighborhood to highlight smaller-scale differences (Table 5). All four urban neighborhoods showed similar T and RH tendencies, with HV locations showing a significantly lower T by as little as 0.061 °C at West Hills and as much as 1.136 °C at Burlington, and a significantly higher RH than MV locations by as much as 4.317% at West Hills. Burlington, Lonsdale, and West Hills all reported a lower HI at the HV location; however, Burlington is the only neighborhood that showed significant difference (mean HI was 1.467 °C lower at the HV location). West Hills exhibited a significant HI of 1.657 °C higher at the HV station than at the MV location.

**Table 5:** Comparison of daily maximum heat index (°C), temperature (°C), and relative humidity (%) between MV and HV locations within each neighborhood. Mean differences are shown. Bolded numbers indicate significant results (*p* < 0.05). Negative numbers indicate the HV location has a lower mean than the MV location in each neighborhood.

Neighborhood	Heat Index	Temperature	Relative Humidity
Lonsdale	−0.021	**−0.235**	**2.409**
West Hills	**1.657**	**−0.061**	**4.317**
Vestal	−0.208	**−0.386**	**1.631**
Burlington	**−1.467**	**−1.136**	**3.171**

Three of the four neighborhoods (Lonsdale, Vestal, and Burlington) showed a reduced HI at the sites with less imperviousness and more tree cover; however, a significantly higher HI was experienced at the West Hills HV station. While the T is significantly lower at the HV location than at the MV location, the amount of variation (0.061 °C) is small whereas the much higher RH at the HV location might help us understand what is happening. The West Hills stations have a relatively similar impervious surface level but a vastly different tree cover level. The increased vegetation at the HV location is likely causing a higher RH because of increased evapotranspiration from the plants, which in turn leads to a higher HI at the HV location.

While the MV (HV) station within the four urban neighborhoods represents what is expected to be the highest (lowest) temperatures, least (most) humid locations, there is a likely a wide range of variability between these two extremes for each neighborhood. Shading from the tree cover and the amount of impervious surfaces will vary throughout each neighborhood. Additionally, although all MV stations have greater impervious surfaces and less tree cover, that does not mean that they are devoid of vegetative effects.

Vegetation types and age [77], neighborhood parks and greenways, building height and density, roadway density, and traffic [78] can all have a large effect on the strength of the UHI for various places within each neighborhood. Parks and greenways represent a prominent method for UHI mitigation and increasing thermal comfort in urban neighborhoods. Increased vegetation results in less energy used for cooling, decreased air pollution, and may potentially reduce greenhouse gasses [79]. However, this specific method of mitigation has associated costs. Aside from the initial costs of purchasing and planting vegetation, upkeep in the form of irrigation from a public water supply, removing dead vegetation, and replanting can keep costs high [79]. Tree planting programs, while effective, are expensive and often distributed to higher-income areas of cities [79]. In locations like urban Phoenix, for example, where water can be scarce, temperatures can be higher by several degrees in areas with lower income because of less tree cover [20].

Additional attempts to alleviate the heat produced and trapped in urban areas have included technologies such as cool-roofs [4,26] and changes to architecture and city planning [4,16,25,80]. However, all of the factors that affect UHI intensity and the human perception of heat will vary by location [4,5,6]. As emphasized by our study of intra-neighborhood variability in impervious surfaces and vegetation cover, a one-size-fits-all prescription to mediate the effects of the UHI may be ineffective and unrealistic, but may assist in urban neighborhood heat reduction and even greenhouse warming if used appropriately [81].

### 3.5. Data Distribution

**Figure 3:**
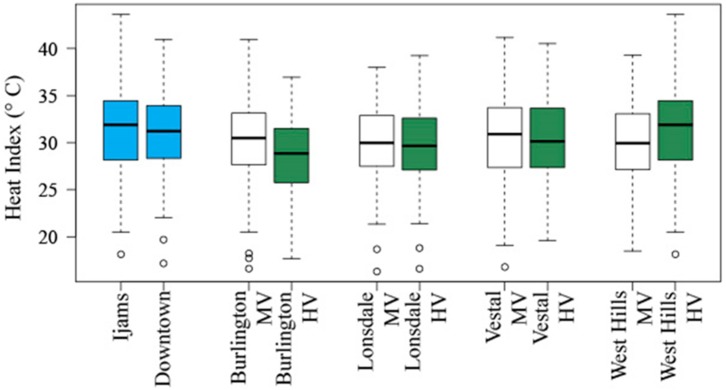
Heat index data range for each station location. Bold horizontal lines indicate median value, boxes represent the upper and lower quartiles of each data set, upper and lower whiskers represent the maximum and minimum values respectively, and open circles represent outliers less than 3/2 times of lower quartile.

The control locations at Ijams and Downtown show a difference in medians with Downtown having a lower median HI than Ijams (Figure 3). The intra-neighborhood differences in the median HI values for Burlington and West Hills show the greatest differences and supports our mean HI *t*-tests described above. The intra-neighborhood differences in median HI values for Lonsdale and Vestal are quite similar, which again is supports our mean HI *t*-test as described above.

Aside from the control locations, percent imperviousness was found to be greatest at the Lonsdale MV and the Vestal MV stations (48.8% and 41.1%, respectively). Despite this, West Hills MV has a similar median HI to the Lonsdale MV station, through the data ranges vary widely, and the Burlington MV station has a similar median HI value to the Vestal MV station. Percent tree cover within the neighborhoods was found to be greatest West Hills HV (60.1%) and Vestal HV (51.3%). Given that we would expect tree cover to result in a higher HI because there will be higher relative humidity, this was only the case for the West Hills HV station.

These results point to the potential that the large scale attributes of imperviousness and tree canopy around the city can affect each location to a greater extent than previously thought. Alternatively, or perhaps in addition to the large scale effect, the impact of imperviousness in some locations (e.g., West Hills) might be more localized than the 100 m radii studied here. The spatial effect of impervious and canopy cover on localized weather conditions is a question that cannot be answered through this study and is subject to further research [82,83]. Convective mixing in each location might additionally reduce the localized cooling effect of radiation blocking and increased humidity from evapotranspiration provided by vegetation, resulting in a more uniform temperature throughout the urban neighborhoods [13,83].

### 3.6. Interacting Effects of Neighborhood and Tree Cover

Three two-way ANOVAs were used to determine how neighborhood and tree cover impacted T, RH, and HI. Results for the T ANOVA are shown in Table 6. Tree cover was the only significant variable, and neighborhood was not a significant contributor to T. It is somewhat surprising that neighborhood was not significant; however, as shown in Table 4, there is significant variability between HV and MV stations in each neighborhood, pointing to tree cover as the main contribution. The lack of a significant interaction between neighborhood and tree cover, as well as no clear neighborhood signal, suggests that more immediate tree cover has a larger influence on T than larger-scale neighborhood characteristics.

**Table 6:** Two-way ANOVA for mean temperature at 1500 LDT for the entire warm season based on neighborhood classification and tree density, including mean-square error (MS), *F* value, and significance. Bold variables are significant (*p* < 0.05).

Variables	MS	*F*-Value	Significance
Neighborhood	21.94	2.210	0.066
**Tree Cover**	88.49	8.913	**0.002**
Neighborhood * Tree	14.69	1.480	0.228

The RH ANOVA results (Table 7) show that tree cover was once again significant, as was neighborhood. The interaction between the two was insignificant. Therefore, tree cover and neighborhood were significant contributors to RH, but the role of tree cover did not change based on the neighborhood. Combining these findings with the results of the T ANOVA suggest that neighborhood-scale decisions, such as tree planting and building density, may play a more consistent role in determining RH than T. The HI ANOVA results (Table 8) are similar to the RH ANOVA, with both neighborhood and tree cover being significant, but not the interaction between the two.

**Table 7:** Two-way ANOVA for mean relative humidity at 1500 LDT for the warm season based on neighborhood classification and tree density, including mean-square error (MS), *F* value, and significance. Bold variables are significant (*p* < 0.05).

Variables	MS	*F*-Value	Significance
**Neighborhood**	1659.1	9.876	**<0.001**
**Tree Cover**	1165.2	6.937	**0.009**
Neighborhood * Tree	48.6	0.289	0.749

**Table 8:** Two-way ANOVA for mean heat index at 1500 LDT for the entire warm season based on neighborhood classification and tree density, including mean-square error (MS), *F* value, and significance. Bold variables are significant (*p* < 0.05).

Variables	MS	*F*-Value	Significance
**Neighborhood**	109.75	5.777	**<0.001**
**Tree Cover**	84.41	4.443	**0.035**
Neighborhood * Tree	41.39	2.179	0.114

### 3.7. Extreme Heat Variability

A two-way ANOVA was used to determine how neighborhood and tree cover impacted HI during the top 10% of the highest HI values from each neighborhood. Results for the top 10% HI ANOVA are shown in Table 9. Both neighborhood and tree cover were significant contributing factors for cross site differences in heat index on the days ranked in the top 10% of HI values in the study region. There was no significant interaction between neighborhood and tree cover found during the highest HI values. The top 10% of HI values have a similar significance pattern to all of our data combined (Table 8), with neighborhood and tree cover contributing to both observed HI across the study period as well as for the top 10% of HI values, but with no interacting effects of neighborhood and tree cover. While neighborhood and tree cover were both significant contributors to HI, it is likely that this contribution is consistent across all neighborhoods.

**Table 9:** Two-way ANOVA for top 10% of heat index values at 1500 LDT based on neighborhood classification and tree density, including mean-square error (MS), *F* value, and significance. Bold variables are significant (*p* < 0.05).

Variables	MS	*F*-Value	Significance
**Neighborhood**	109.75	5.777	**<0.001**
**Tree Cover**	84.41	4.443	**0.035**
Neighborhood * Tree	41.39	2.179	0.114

## 4. Conclusions

While temperature is often the focus of UHI studies, this work addresses the need to include humidity to better understand local heat exposure. By comparing locations with different levels of imperviousness and tree cover, it is clear that there are competing factors that influence the HI of an area. A location with greater impervious surfaces and little vegetation will likely experience a greater maximum temperature. Meanwhile, a location with expansive tree cover and vegetated surfaces will likely have higher humidity.

This work additionally emphasizes the importance of using smaller-scale data, such as at the neighborhood-level, to determine which neighborhoods and corresponding socioeconomic groups experience the most influence of relative humidity on high temperatures within a medium-sized city. Studies conducted in larger cities, such as Phoenix, have shown that predominately white neighborhoods with a higher mean income experience less heat stress [11]. However, of the four Knoxville neighborhoods studied here, the neighborhood with the greatest HI exposure is the wealthiest and is predominantly White. This may seem somewhat encouraging for the other neighborhoods; however, it is likely that the lack of resources to cope with HI will counteract any difference in exposure. Complete understanding of risk and vulnerability to heat exposure will require these results to be paired with further quantitative and qualitative data collected directly from urban residents in diverse neighborhoods.

Understanding how UHI impacts residents through HI variability in a medium-sized city on a neighborhood-level is a timely and critical issue and can help inform heat mitigation efforts (e.g., heat advisory warning systems, neighborhood heat exposure education for both awareness of heat exposure consequences and personal mitigation) for other similar-sized cities. It is important to understand that other factors exist that were not addressed in this study. We used HI with the assumption that this index would give a better understanding of the level of the influence of humidity on temperature in different urban settings. Given this, we did not consider individually experienced temperatures or the human energy budget as our data did not allow for this scale of analysis. Radiative effects that are influenced by shading from vegetation, such as blocking of solar radiation, will influence the human energy budget by reducing the radiative heat exchange between the environment and the person by as much as 30% [84]. Increased wind speed can also account for cooling of the individual and can somewhat offset the effects of short- and long- wave radiation fluxes and temperature on the human energy budget [85,86,87].

Additionally, while neighborhood-level data show a much more complex relationship between urban neighborhoods and the urban heat island than previously considered, access to resources such as air conditioners, residential building height and structure, the amount of time spent indoors *versus* outdoors, and the resiliency and vulnerability of people living in each neighborhood were not considered, but could affect how residents experience heat during the warm season in Knoxville. Further research, including resident interviews and individual or household surveys, could help to shed light on these factors and further inform heat mitigation and land cover management.
